# Effects of music-based occupational therapy activities on attention executive functions in children with attention deficit and hyperactivity disorder

**DOI:** 10.1371/journal.pone.0349284

**Published:** 2026-05-18

**Authors:** Ibrahim Erarslan, Miray Budak, Devrim Tarakci

**Affiliations:** 1 Department of Occupational Therapy, Institute of Health Sciences, Istanbul Medipol University, Istanbul, Turkey; 2 Center for Molecular and Behavioral Neuroscience, Rutgers University, Newark, New Jersey, United States of America; Taipei Veterans General Hospital, TAIWAN

## Abstract

**Background:**

Attention Deficit/Hyperactivity Disorder (ADHD) is a multifactorial neurodevelopmental disorder characterized by cognitive, motor, sensory, emotional, and behavioral difficulties. This disorder may lead to impairments in various executive functions, such as sustained attention, planning and organizing, and impulse control. Occupational therapy interventions offer strategies aimed at enhancing attention, executive functions, and motor skills in individuals with ADHD, while also providing guidance and support to caregivers throughout the process. Caregivers of children with ADHD play a vital supportive role and are required to manage caregiving demands through appropriate and healthy care strategies.

**Objective:**

The aim of this study was investigate the effect of music-based occupational therapy activities on attention and executive functions in children diagnosed with ADHD, compared with structured occupational therapy interventions, as well as to examine changes in caregiver burden.

**Materials and methods:**

A total of 39 children aged between 5 and 12 years (N = 39) participated in the study. Participants were randomly assigned into two groups: music-based occupational therapy (n = 19) and structured occupational therapy (n = 20). The music-based occupational therapy group received sessions involving harmonica and drum-based occupational therapy activities, while the structured occupational therapy group received conventional structured occupational therapy interventions. Both interventions were administered once a week for six weeks, with each session lasting 45 minutes. Children’s attention levels were assessed using the DSM-V Level-2 Inattention Scale, executive function skills were evaluated with the Behavior Rating Inventory of Executive Function–Child Version (BRIEF), and caregiver burden was measured using the Zarit Burden Interview, both at baseline and after the intervention period.

**Results:**

Pre- and post-intervention assessments revealed improvements in both groups in terms of attention levels (F_(1, 37)_ = 9.675, *η*² = .207, p = .004), executive functions (F_(1, 37)_ = 2.506, *η*² = .063, p = .122), and caregiver burden (F_(1, 37)_ = 0.002, *η*² = .000, p = .967). When comparing time-group interactions, it was observed that music-based occupational therapy activities had a more beneficial effect, particularly on attention levels, in children with ADHD compared to structured occupational therapy.

**Conclusion:**

Overall, music-based occupational therapy activities demonstrated positive effects on attention levels, executive functions, and caregiver burden in children with ADHD. Future studies are encouraged to incorporate a broader range of musical instruments into occupational therapy intervention plans, aligned with specific therapeutic goals. It is considered important for therapists to develop strategies that tailor the rhythm, melody, and harmony of musical instruments according to the physical, emotional, and cognitive needs of individuals.

## 1. Introduction

Attention Deficit Hyperactivity Disorder (ADHD) is a complex neurodevelopmental and neuropsychiatric disorder characterized by core symptoms such as distractibility, difficulty sustaining attention, irritability, impulsivity, and hyperactivity [1–3]. ADHD affects approximately 3%–7% of the pediatric population worldwide, with the symptoms typically emerging before the age of seven and persisting for at least six months.

Children with ADHD experience significant difficulty focusing and sustaining attention and inhibiting impulsive behaviors. Observable behaviors—such as interrupting others, difficulty waiting for one’s turn, the tendency to respond prematurely, and the inability to delay gratification— are hallmark indicators of impulse control deficits [2]. Attention, defined as the cognitive and behavioral process of selectively concentrating on specific stimuli while ignoring others, plays a critical role in organizing behavior and guiding goal-directed actions [4]. It functions as a neural filter, managing sensory input and shaping the organism’s interaction the environment [5]. Attention and concentration are foundational to executive functions, which encompass a set of higher-order cognitive skills essential for self-regulation, problem-solving, and adaptive functioning [6,7]. Tunç and Melekoğlu (2024) demonstrated that students with ADHD experience persistent inattention, disruptive behavior, aggression, working memory impairments, and difficulties in planning and organization, primarily due to neuropsychological deficits, which in turn may lead to academic underperformance [8,9]. Executive function impairments occur in approximately 80% of children with ADHD, at least once during their developmental trajectory [10]. The ability to employ cognitive strategies related to executive function is highly critical for effective task engagement and is primary target of occupational therapy interventions [11]. Evaluating performance in complex, multi-step tasks that require integration of executive functions may provide a better understanding of activity performance deficits in at-risk populations [11,12].

Caregivers of children with ADHD often encounter a range of challenges, including limited understanding of the disorder, insufficient time for self-care, and physical, emotional, social, and economic difficulties [13]. Occupational therapists, therefore, emphasize family-centered approaches, actively involving caregivers in therapy planning and implementation. They provide structured guidance to families through home-based programs that reinforce the child’s therapeutic gains [14,15]. Daly et al. (2022) highlighted that family engagement fosters a deeper understanding of the child’s needs and facilitates meaningful participation in daily routines [16].

Occupational therapy interventions for ADHD commonly address deficits in cognitive, motor, emotional, behavioral, and sensory domains that impact a child’s ability to engage in activities of daily living (ADL) [17]. Therapists develop individualized treatment plans following detailed assessments of how these impairments affect occupational performance. Among emerging interventions, music-based activities grounded in occupational therapy principles have gained increasing attention. Music by engaging auditory, motor, emotional, and cognitive systems simultaneously, serves as a powerful therapeutic modality. It has been shown to enhance motivation, self-esteem, and social engagement, particularly in group-based sessions. Moreover, numerous studies have demonstrated positive correlations between musical activities and improvements in cognitive functions, including attention and executive processes [18,19]. Techniques such as rhythmic stimulation, vocalization, music listening, and improvisation are used therapeutically to target specific functional domains [20].

Given the multidimensional nature of ADHD-related impairments, music and rhythm-based interventions may enhance traditional occupational therapy approaches by improving attention regulation and executive function. This study investigates the effect of music-based occupational therapy interventions on attention and executive function in children diagnosed with ADHD, while also examining changes in caregiver burden. These findings aim to provide original empirical support fort he integration of music-based strategies within occupational therapy, offering a dual benefit for both children and their caregivers.

## 2. Materials and methods

### 2.1. Study design

This study was designed as a single-blind (evaluator-blinded), randomized controlled clinical trial, conducted between April 2022 and April 2023 at three locations: the Pediatric Rehabilitation Laboratory of the Department of Occupational Therapy at Istanbul Medipol University, Gülseren Özdemir Special Education and Practice School, and Basamak Special Education and Rehabilitation Center. Ethical approval was granted by the Non-Invasive Clinical Research Ethics Committee of Istanbul Medipol University on March 24, 2022 (Approval No: E-10840098-772.02-2028). The study protocol was registered retrospectively on ClinicalTrials.gov with the identifier (NCT07253558). The delay in registration occurred due to operational requirements and unforeseen scheduling constraints during the early phase of participant recruitment. Despite the retrospective registration, the study was conducted in full compliance with internationally recognized ethical principles. Because the participants were children, the study was conducted with the consent of the children’s parents. The study was conducted using a voluntary consent form, and written informed consent was obtained from the parents. Written informed consent was obtained from all parents or legal guardians prior to participation. Participants were assigned to two groups using stratified randomization via the website Random.org: the music-based occupational therapy group (n = 19) and the structured occupational therapy group (n = 20). Both intervention groups participated in weekly 45-minute sessions over a period of six weeks. The music-based group received sessions incorporating musical musical instruments and structured music-based activities, while the structured therapy group received conventional, non-musical occupational therapy interventions targeting ADHD-related functional impairments.

Although the study protocol was prepared before the initiation of the trial, there was an administrative delay in the final registration process within the (ClinicalTrials.gov) system. Registration was finalized after recruitment had commenced. The authors strictly adhered to the original study design and declare that all data collection and analysis were performed in accordance with the initial ethical guidelines and intended methodology. The authors confirm that all ongoing and related trials for this drug/intervention are registered.

A minimum anonymized dataset supporting the findings of this study has been uploaded to the Aperta repository to ensure transparency and reproducibility. The data can be accessed via this Digital Object Identifier (DOI): https://doi.org/10.48623/aperta.286776

### 2.2. Study population

**Fig 1:**
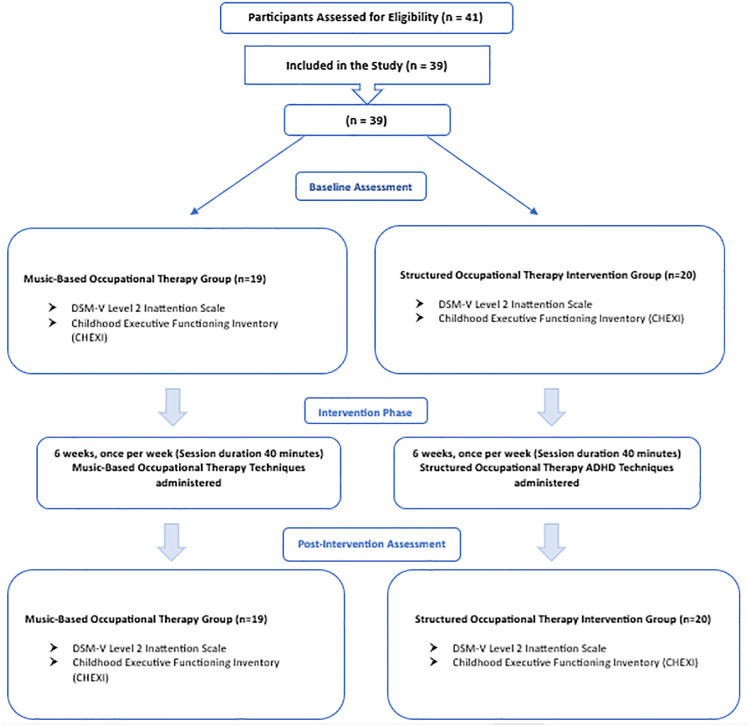
Design and flow chart of the study.

A total of 39 children diagnosed with ADHD, aged the provision of written informed consent.and residing in Istanbul, were enrolled in the study following the provision of written informed consent. Although the initial target sample size was 41, two caregivers initially assigned to the music-based occupational therapy group were excluded from the study—one due to a score of 33 on the Beck Depression Inventory (BDI), and the other due to anticipated travel outside the city during the intervention period. The inclusion criteria for the study were as follows: having a clinical diagnosis of ADHD, being between 5 and 12 years of age, residing in Istanbul, the caregiver scoring 21 or above on the Parenting Stress Index (PSI), and the caregiver being between 18 and 65 years of age. Exclusion criteria included: having a comorbid diagnosis in addition to ADHD, experiencing a cardiopulmonary condition within the last three months, prior experience in playing a musical instrument by the child, and the caregiver scoring 31 or above on the Beck Depression Inventory (BDI). Eligibility was determined based on predefined inclusion and exclusion criteria, and The participant flow diagram is presented in Fig 1. as they were responsible for completing the study assessments. The participant flow diagram is presented in Fig 1.

The required sample size was calculated using the G*Power version 3.1 [21], targeting a statistical power of 95% (α = 0.05, 1-β = 0.95), a critical F-value of 4.195, and an effect size of 0.35. This yielded a required sample size of 30 participants for a two-group, two-timepoint “ANOVA: Repeated Measures, within-between interaction” design.

### 2.3. Intervention protocol

#### 2.3.1. Structured occupational therapy intervention.

Participants in the active control group received structured, evidence-based occupational therapy interventions tailored to ADHD. Sessions were conducted once weekly for six weeks (45-minutes each). This protocol included structured pediatric occupational therapy techniques such as sensory integration-based activities, caregiver guidance on activities that enhance environmental awareness in the home setting, training and recommendations regarding appropriate games and toys to be used at home, turn-taking and rule-following skills during play, and ergonomic adjustments in the home environment for children with ADHD (e.g., removing scattered toys from the floor).

#### 2.3.2. Music-based occupational therapy intervention.

Participants in the experimental group received music-based occupational therapy interventions in 45-minute sessions, once a week, for a total duration of six weeks. In addition to the interventions and applications included in the structured protocol, this intervention incorporated the use of musical instruments, specifically drums and harmonicas. These instruments were selected for their ability to enhance fine motor skills, provide oral-motor sensory input, require diaphragmatic breathing coordination, promote bimanual activity performance, and incorporate rhythmic components. The use of these materials was facilitated either through shared attention between the therapist and the child or through individually guided practice based on verbal prompts provided to the child. These instruments were integrated into sensory-based play activities grounded in occupational therapy principles to support therapeutic outcomes. Musical instruments were used alongside sensory tools such as a ball pit, platform swing, Lycra hammock, trampoline, and play tunnel. In addition to targeting attention and executive functions, the intervention also aimed to support the development of play skills, self-regulation, turn-taking abilities, fine motor skills, motor coordination, and structured play performance.

### 2.4. Outcome measurements

**Neuropsychometric tests:** For the evaluation of caregivers’ cognitive functioning one of the inclusion criteria of the study the Montreal Cognitive Assessment (MoCA) was used. To assess caregivers’ depression levels, which also constituted an inclusion criterion, the Beck Depression Inventory (BDI) was administered. To evaluate attention levels, the DSM-5 Level 2 Inattention Scale was used. The Childhood Executive Functioning Inventory – Parent/Teacher Form (CHEXI) was employed to assess children’s executive function skills.

To assess caregiver stress levels and caregiver burden, the Zarit Burden Interview (ZBI) was utilized.

**MoCA:** The scale was developed by Nasreddine et al. with the aim of identifying individuals with mild cognitive impairment as well as those with normal cognitive functioning. The Turkish adaptation of the scale was conducted by Selekler et al. [22]. The scale includes subdomains such as visuospatial/executive functions, naming, attention, language, abstract thinking, memory, and orientation. The maximum score that can be obtained from the scale is 30, while the minimum score is 0. A score of 21 or above is considered within the normal range [23].

**Beck depression scale (BDI):** The Beck Depression Inventory was developed by Beck et al. in 1974 to quantify the severity of depressive symptoms. It is a self-report assessment tool consisting of 21 items measuring somatic, emotional, and cognitive symptoms commonly associated with depression. The maximum possible score is 63, with higher total scores indicating greater severity of depressive symptoms. The Turkish validity and reliability of the inventory were established by Hisli [24].

**DSM-V level 2 inattention scale:** The DSM-5 Level 2 Inattention Scale includes an 8-item parent-report form designed for children aged 6–17 years. This form is completed by a parent, guardian, or caregiver and evaluates the child’s inattention symptoms over the previous seven days. It is intended to be filled out by the caregiver prior to the clinical interview with the clinician. In this scale, the caregiver rates the child’s level of inattention for each item based on observations from the previous week. The total score ranges from 0 to 24, with higher scores indicating a greater severity of inattention. The Turkish version of the DSM-5 Level 2 Inattention Scale has been validated and tested for reliability by Ömer Aydemir and colleagues [25].

**Childhood executive functioning inventory – Parent/teacher form (CHEXI):** The Childhood Executive Functioning Inventory (CHEXI) was developed in 2008 by Lisa B. Thorell and Lilianne Nyberg as a measurement tool specifically focused on executive functions. The Turkish translation and adaptation of the inventory were carried out by Ezgi Kayhan in 2010 [26]. CHEXI is suitable for use with children aged 4–12 years and consists of 26 items divided into four subscales: Working Memory (9 items) Planning (6 items) Inhibition (6 items) Regulation (5 items) Each item is rated on a 5-point Likert scale based on how accurately the statement applies to the child (1: not at all true, 5: completely true) [27].

Although the CHEXI is traditionally conceptualized as a multidimensional instrument consisting of four subscales (working memory, planning, inhibition, and regulation), in the present study a total score was used to capture overall executive functioning difficulties. This approach has been applied in prior research when a global index of executive dysfunction is of interest. However, we acknowledge that the use of a total score may obscure subdomain-specific effects. Future studies are encouraged to examine subscale-level outcomes to enhance construct validity and interpretability.

**Zarit burden interview (ZBI):** The Zarit Burden Interview (ZBI) was originally developed in 1980 by Zarit and colleagues to assess the burden experienced by caregivers of individuals with dementia. The scale evaluates the challenges and stressors faced by caregivers providing care to individuals in need of support. The ZBI consists of 22 items and uses a 5-point Likert-type scale, ranging from “never” to “nearly always,” with response options scored between 1 and 5. Higher total scores indicate greater perceived caregiver burden. In Türkiye, the validity and reliability study of the scale was conducted by Özlü and colleagues with caregivers of 100 individuals diagnosed with schizophrenia [28].

### 2.5. Statistical analysis

Statistical analyses were conducted using IBM SPSS Statistics for Windows, Version 25.0. Descriptive statistics were presented as mean ± standard deviation for continuous variables and frequencies and percentages for categorical variables. The normality of data distribution was assessed using the Kolmogorov–Smirnov test. Categorical variables were analyzed using the Chi-square test. For comparisons of continuous variables between groups at baseline, the Independent Samples t-test or the Mann–Whitney U test was used, depending on data distribution. Within-group time-dependent differences and between-group time*group interactions were examined using Two-Way Repeated Measures ANOVA. A significance level of p < 0.05 was considered for all statistical tests.

## 3. Results

### 3.1. Demographics

Demographic characteristics of the 39 children with ADHD (19 girls) included in the study are presented in Table 1. All caregiver participants were mothers, which is culturally normative in Türkiye, where mothers are typically the primary caregivers of children. Due to privacy concerns, caregivers declined to provide detailed demographic information. There were no statistically significant differences between the groups in terms of children’s age or sex (p > 0.05) (Table 1).

**Table 1:** Demographic characteristics.

		Structured Occupational Therapy (n = 20)	Music-Based Occupational Therapy (n = 19)	Between-Group Differences
Age (Mean± SD)		7.60 ± 1.53	7.11 ± 1.15	*t*_*37*_ *=-1.134, p=0.264*
Gender	Female n (%)	10 (50)	9 (47.4)	*X*_*1*_ *= 0.027, p = 0.869*
	Male (n/%)	10 (50)	10 (52.6)	

### 3.2. Within-group comparisons

#### 3.2.1. Music-based occupational therapy group.

Repeated measures analysis for the music-based occupational therapy group is presented in Table 2. Statistically significant within-group improvements were observed from pre- to post-intervention in attention (DSM-V Level 2 Inattention Scale), executive functions (CHEXI), and caregiver burden (ZBI) (p < .001 for all). These findings indicate improvements in attention and executive functioning scores, along with a reduction in caregiver burden following the intervention.

**Table 2:** Time-dependent changes in the music-based occupational therapy group.

Music-Based Occupational Therapy (n = 19)		Pre-Treatment	Post-Treatment	Mean Difference	Confidence Interval (Lower/ Upper)	Statistics
		Mean ± SD	Mean ± SD			
**Attention**	DSM-V Level 2 Inattention Scale	2.36 ± 0.49	1.15 ± 0.37	−1.211	−29.386/-21.351	*F*_*(1,37)*_ *=70.015,* *η* ^*2*^ =.795, p=<.001*
**Executive Functions**	CHEXI	103.63 ± 6.23	78.26 ± 8.33	−25.368	−4.890/11.942	*F*_*(1,37)*_*=176.018, η*^*2*^ *=* .907, p=<.001*
**Caregiver Burden**	ZBI	63.47 ± 6.69	51.74 ± 5.59	−11.737	−14.395/-9.078	*F*_*(1,37)*_*= 86.020,* *η*^*2*^*=*.827, p=<.001*

SD: standard deviation; CHEXI: Childhood Executive Functioning Inventory; ZBI: Zarit Burden Interview.

#### 3.2.2. Structured occupational therapy intervention group.

Repeated measures analysis for the structured occupational therapy group is presented in Table 3. Statistically significant within-group improvements were observed across all outcome measures from baseline to post-intervention (p < .05).

**Table 3:** Time-dependent changes in the structured occupational therapy group.

Structured Occupational Therapy (n = 20)		Pre-Treatment	Post-Treatment	Mean Difference	Confidence Interval (Lower/ Upper)	Statistics
		Mean ± SD	Mean ± SD			
**Attention**	DSM-V Level 2 Inattention Scale	2.45 ± 0.51	1.80 ± 0.41	−0.650	−0.879/-0.421	*F*_*(1,37)*_*=70.015, η*^*2*^ *= .650* p=<.001*
**Executive Functions**	CHEXI	100.60 ± 5.96	79.95 ± 8.02	−20.650	−25.394/-15.906	*F*_*(1,37)*_*=35.286, η*^*2*^ *= .814* p=<.001*
**Caregiver Burden**	ZBI	59.9 ± 5.93	48.30 ± 6.50	−11.650	−15.063/-8.237	*F*_*(1,37)*_*= 51.036, η*^*2*^ *= .729* p=<.001*

SD: Standard Deviation CHEXI: Childhood Executive Functioning Inventory ZBI: Zarit Burden Interview.

### 3.3. Between-group differences

Between group comparisons of pre- and post-intervention scores for the DSM-V Level 2 Inattention Scale, CHEXI, and the ZBI are presented in Table 4. A statistically significant group difference was observed only in the DSM-V Level 2 Inattention Scale, favoring the music-based occupational therapy group (p < .05). No statistically significant differences were found between groups for the CHEXI or ZBI scores (p > 0.05). These findings suggest that while both interventions were effective within groups, music-based occupational therapy group resulted in greater improvements specifically in attention regulation as measured by the DSM-V Level 2 Inattention Scale.

**Table 4:** Between-group differences.

	Pre-Treatment			Post-Treatment		Statistics
	Mean ± SD	Mean ± SD		Mean ± SD	Mean ± SD	
**DSM-V Level 2 Inattention Scale**	2.36 ± 0.49	2.45 ± 0.51	0.182	1.15 ± 0.37	1.80 ± 0.41	*F (*_*1,37*_*)=9.675, η*^*2*^ *= .207* p < .0.004*
**CHEXI**	103.63 ± 6.23	100.60 ± 5.96	0.647	78.26 ± 8.33	79.95 ± 8.02	*F (*_*1,37*_*)=2.506, η*^*2*^ *= .063* *p = .122*
**ZBI**	63.47 ± 6.69	59.95 ± 5.93	0.913	51.74 ± 5.59	48.30 ± 6.50	*F (*_*1,37*_*)=*0.002, *η*^*2*^ *= .000* *p = .967*

SD: Standard Deviation CHEXI: Childhood Executive Functioning Inventory ZBI: Zarit Burden Interview.

## 4. Discussion

This study investigated the comparative effects of music-based occupational therapy interventions and structured occupational therapy interventions on attention, executive functions, and caregiver burden in children with ADHD. The findings demonstrated that both intervention protocols led to significant improvements in attention, executive functions, and caregiver burden. Notably, children in the music-based occupational therapy group exhibited significantly greater gains in attention compared to the structured occupational therapy group.

ADHD is a highly prevalent neurodevelopmental disorder that significantly disrupts academic achievement, social functioning, and overall quality of life in children and adolescents [29]. Central to its clinical presentation are impairments in executive functioning and attention regulation. Occupational therapy interventions commonly target deficits in cognitive, sensory, motor, and behavioral domains, aiming to improve daily occupational performance [17]. In the cognitive domain, therapists assess the functional implications of executive dysfunctions and design individualized interventions accordingly.

Previous literature supports the role of occupational therapy in enhancing attention and executive functions in children with ADHD. Likewise, studies have demonstrated that occupational therapy approaches incorporating sensory integration and caregiver engagement are effective in reducing behavioral complaints reported by both families and educators [30,31]. For example, Gurlek and Bumin (2024) reported that a 12-session occupational therapy intervention yielded significant improvements in activity performance and daily executive functioning in children with ADHD [32].

In our study, 39 children with ADHD were randomly assigned to either a music-based or structured occupational therapy group. Both interventions were implemented in clinical and home settings and incorporated motor, sensory, cognitive, and behavioral components. However, the music-based intervention included the targeted use of musical instruments—such as drums and harmonicas—within therapeutic play. These instruments were selected for their potential to enhance fine motor coordination, provide sensory input, and engage attention through rhythmic structure and auditory-motor integration. Findings indicated that both groups improved significantly in executive functioning, attention, and caregiver burden. However, the music-based group showed significantly greater improvement in attention, a core deficit in ADHD. We attribute this enhanced effect to the engaging, motivating, and structured nature of music-based activities, which likely increased participation and focus during sessions. Additionally, the incorporation of musical instruments into home-based assignments may have reinforced therapeutic gains outside the clinical setting [33,34].

Music-based occupational therapy activities provide a multimodal therapeutic framework that supports emotional regulation, cognitive engagement, and social participation. Previous studies support these findings. Martínez-Vérez et al. (2024) reported significant improvements in communication, behavior, and emotional skills following musical activity interventions in children with ADHD [35]. Similarly, Cagape (2024) found that musical activities reduced anxiety and enhanced attention and emotional regulation, thereby increasing attention levels in children with ADHD [36]. These findings collectively support the growing evidence base that music-enhanced occupational therapy can optimize treatment outcomes for children with neurodevelopmental disorders.

In our study, families in both intervention arms reported positive feedback and observable improvements in daily functioning. However, families in the music-based group noted higher engagement and motivation in their children, potentially contributing to the superior gains in attention. The structured yet enjoyable natüre of musical activities likely enhanced sustained participation, making therapy both effective and meaningful. The use of diverse musical materials rather than traditional tools enabled greater creativity, sensory integration, and emotional expression. Incorporating music into occupational therapy offers a unique opportunity to develop customized, child-centered intervention plans, thereby broadening the scope of therapeutic strategies available for children with ADHD.

It is important to note that several within-group effect sizes observed in this study were large (η² > .80). While these findings may indicate substantial changes over time, such large effect sizes should be interpreted with caution, particularly given the relatively small sample size and short intervention duration. These values may partly reflect shared method variance associated with caregiver-reported outcomes, as well as increased caregiver engagement or expectancy effects. Therefore, although the results are promising, they should be considered preliminary and require replication in larger, more rigorously controlled studies.

### 4.1. Limitations

This study has several limitations that should be considered when interpreting the findings.

*First,* all primary outcome measures relied on caregiver-reported assessments. Because caregivers were not blinded to group allocation and were actively involved in the intervention process, the potential for expectancy and reporting bias cannot be ruled out. This may have led to an overestimation of treatment effects, particularly in subjective domains such as attention and executive functioning.

*Second,* caregiver engagement, while clinically meaningful, may have influenced perceptions of improvement independent of objective change. Therefore, the findings should be interpreted with caution. Future studies should incorporate multi-informant assessments (e.g., teacher reports), as well as objective or performance-based neuropsychological measures, to reduce bias and strengthen the validity of outcomes.

*Third*, the use of a total CHEXI score rather than subscale-level analyses may have limited the ability to detect domain-specific effects within executive functioning.

*Finally,* the lack of caregiver demographic data represents an additional limitation. All participating caregivers (mothers) declined to disclose this information, which, although culturally understandable in the context of Türkiye, precluded subgroup analyses that could have clarified the impact of caregiver-related factors on outcomes. Moreover, the inclusion of only mothers may limit the generalizability of the findings to more diverse caregiver populations.

## 5. Conclusions

This study provides preliminary evidence that music-based occupational therapy is an effective and engaging intervention for improving attention and executive functioning in children with ADHD, while also reducing caregiver burden. These findings highlight the therapeutic potential of integrating structured musical activities into occupational therapy to enhance cognitive outcomes and family well-being. Further research with larger samples and longer follow-up is warranted to confirm and extend these results, and to clarify the mechanisms through which music exerts its therapeutic effects.

## Supporting information

S1 File CONSORT checklist. (DOCX)

S2 File Protocol. (DOCX)
