# Supplementary material for: Effects of music-based occupational therapy activities on attention executive functions in children with attention deficit and hyperactivity disorder
Source: PLoS One. 2026 May 18;21(5):e0349284. doi: 10.1371/journal.pone.0349284 (PMC13183191; doi:10.1371/journal.pone.0349284)
Supplement: S2 File — (DOCX) [file pone.0349284.s002.docx]

**S1_Protocol: Clinical Trial Protocol**

**Title:** Effects of music-based occupational therapy activities on attention executive functions in children with attention deficit and hyperactivity disorder

**ClinicalTrials.gov ID:** NCT07253558

**1. Administrative Information**

- **Trial Registration:** Registered at ClinicalTrials.gov (NCT07253558). Although registered retrospectively due to administrative constraints, the study strictly followed the initial design approved by the ethics committee.
- **Ethical Approval:** Approved by the Non-Invasive Clinical Research Ethics Committee of Istanbul Medipol University (March 24, 2022; Approval No: E-10840098-772.02-2028).
- **Data Availability:** A minimum anonymized dataset supporting the findings has been uploaded to the Aperta repository (DOI: 10.48623/aperta.286776).

**2. Introduction and Objectives**

The primary objective of this study is to investigate the effects of music-based occupational therapy activities on attention and executive functions in children with attention deficit and hyperactivity disorder (ADHD) and to evaluate the associated changes in caregiver burden.

**3. Study Design and Setting**

- **Design:** Single-blind (evaluator-blinded), randomized controlled clinical trial.
- **Setting:** Pediatric Rehabilitation Laboratory of the Department of Occupational Therapy at Istanbul Medipol University, Gülseren Özdemir Special Education and Practice School, and Basamak Special Education and Rehabilitation Center.

**4. Eligibility Criteria**

**4.1. Inclusion Criteria (Children)**

- Clinical diagnosis of ADHD.
- Between 5 and 12 years of age.
- Residing in Istanbul.
- No prior experience in playing a musical instrument.

**4.2. Caregiver Inclusion/Exclusion Criteria**

- **Inclusion:** Aged 18–65 years; Parenting Stress Index (PSI) score ≥ 21.
- **Exclusion:** Beck Depression Inventory (BDI) score ≥ 31; presence of comorbid diagnoses in the child; recent cardiopulmonary conditions (within 3 months).

**5. Randomization and Blinding**

- **Allocation:** Participants were assigned to two groups using stratified randomization via Random.org: the music-based occupational therapy group (n = 19) and the structured occupational therapy group (n = 20).
- **Blinding:** The study was conducted as an evaluator-blinded trial. Outcome assessments were performed by therapists who were blinded to the group assignments.

**6. Intervention Protocols**

Both groups participated in weekly 45-minute sessions over a period of six weeks.

**6.1. Structured Occupational Therapy Intervention**

This protocol included evidence-based pediatric occupational therapy techniques such as sensory integration-based activities, caregiver guidance on environmental awareness, training on rule-following skills during play, and ergonomic home adjustments.

**6.2. Music-Based Occupational Therapy Intervention**

In addition to the structured occupational therapy protocol, this intervention incorporated musical instruments (drums and harmonicas) integrated into sensory-based play. Activities focused on fine motor skills, oral-motor sensory input, diaphragmatic breathing coordination, and rhythmic shared attention.

**7. Outcome Measurements**

- **Attention:** DSM-5 Level 2 Inattention Scale.
- **Executive Functions:** Childhood Executive Functioning Inventory (CHEXI) - Parent/Teacher Form.
- **Caregiver Burden:** Zarit Burden Interview (ZBI).
- **Baseline Assessments:** Montreal Cognitive Assessment (MoCA) and Beck Depression Inventory (BDI).

**8. Sample Size and Statistical Power**

The sample size was calculated using G*Power version 3.1. Targeting a statistical power of 95% (α = 0.05, 1-β = 0.95), and an effect size of 0.35, a minimum of 30 participants was required for the "ANOVA: Repeated Measures, within-between interaction" design.

**9. Statistical Analysis**

Data were analyzed using IBM SPSS Statistics (Version 25.0). Normality was assessed via the Kolmogorov–Smirnov test. Within-group and between-group differences were examined using Two-Way Repeated Measures ANOVA. A significance level of p < 0.05 was applied.
